# Germ-Line Recombination Activity of the Widely Used hGFAP-Cre and Nestin-Cre Transgenes

**DOI:** 10.1371/journal.pone.0082818

**Published:** 2013-12-09

**Authors:** Jiong Zhang, Pavel Dublin, Stephanie Griemsmann, Alexandra Klein, Ralph Brehm, Peter Bedner, Bernd K. Fleischmann, Christian Steinhäuser, Martin Theis

**Affiliations:** 1 Institute of Cellular Neurosciences, Medical Faculty, University of Bonn, Bonn, Germany; 2 Institute of Physiology I, Medical Faculty, Life and Brain Center, Bonn, Germany; 3 Anatomisches Institut, Tierärztliche Hochschule Hannover, Hannover, Germany; University of Tokyo, Japan

## Abstract

Herein we demonstrate with PCR, immunodetection and reporter gene approaches that the widely used human Glial Fibrillary Acidic Protein (hGFAP)-Cre transgene exhibits spontaneous germ-line recombination activity in leading to deletion in brain, heart and tail tissue with high frequency. The ectopic activity of hGFAP-Cre requires a rigorous control. We likewise observed that a second widely used nestin-Cre transgene shows germ-line deletion. Here we describe procedures to identify mice with germ-line recombination mediated by the hGFAP-Cre and nestin-Cre transgenes. Such control is essential to avoid pleiotropic effects due to germ-line deletion of loxP-flanked target genes and to maintain the CNS-restricted deletion status in transgenic mouse colonies.

## Introduction

The Cre/loxP system of site-specific recombination is a powerful tool to achieve cell-type restricted deletions in the mouse [1], [2], even though it has certain limitations [3]. These include detrimental effects of Cre overexpression [4]–[6], spontaneous ectopic Cre activity [7], [8] and spontaneous loss of Cre activity [9], [10]. For gene deletion in the CNS, a Cre transgene driven by promoter elements of the human glial fibrillary acidic protein (hGFAP-Cre) has been used extensively [11]. We and others have used this hGFAP-Cre transgene to study the role of astrocytic gap junction proteins [12]–[19]. In addition, also the nestin-Cre transgene [20] is widely used to delete the major astrocytic gap junction protein, connexin43 (Cx43) [21] or to replace the wildtype (WT) version with mutant versions of Cx43 [22]. Besides Cx43, a second gap junction protein, i.e. Cx30, is expressed in astrocytes [23]. In order to study the role of astrocytic connexins in brain physiology, we and others previously generated mice doubly deficient for Cx30 and Cx43 by interbreeding Cx30 deficient mice [24] with mice lacking Cx43 in astrocytes [12]. These double deficient Cx30^-/-^; Cx43^fl/fl^: hGFAP-Cre mice (DKO mice) showed complete absence of tracer coupling following biocytin filling of astrocytes, impaired spatial potassium buffering, increased neuronal excitability and a propensity for epileptiform activity in the hippocampus [14], [18], as well as deficits in adult neurogenesis [16]. Recently, we observed spontaneous loss of hGFAP-Cre activity in our mouse colony and developed control procedures to maintain stably active hGFAP-Cre in our animal facility [10]. We now report that there is besides the well-known tissue specificity also unexpectedly germ-line activity of hGFAP-Cre as well as of nestin-Cre, two widely used transgenes for astrocyte-directed gene deletion. Ectopic, global deletion of floxed genes occurs with high frequency, which requires an even more rigorous control. We here outline procedures to detect and minimize Cre-mediated germ-line deletion that are essential to avoid unwanted global deletion of floxed alleles and to maintain the CNS-restricted deletion status of floxed alleles in transgenic mouse colonies.

## Results

### Germ-line hGFAP-Cre activity in Cx43 conditional knock-out mice assessed by tail-tip PCR

In an attempt to generate mice in which Cx43 still mediates gap junctional coupling but no longer adhesive interactions via its C-terminal tail (see [23]), we raised Cx43^fl/K258Stop^: hGFAP-Cre mice carrying one Cx43^K258Stop^ allele (coding for carboxyl-terminally truncated Cx43) [25] and a Cx43^fl^ allele [26] which is deleted in the CNS by virtue of the hGFAP-Cre transgene [12]. In order to express solely mutant connexins we crossed these mice with Cx30 KO mice to obtain Cx30^-/-^; Cx43^fl/K258Stop^: hGFAP-Cre mice.

**Figure 1:**
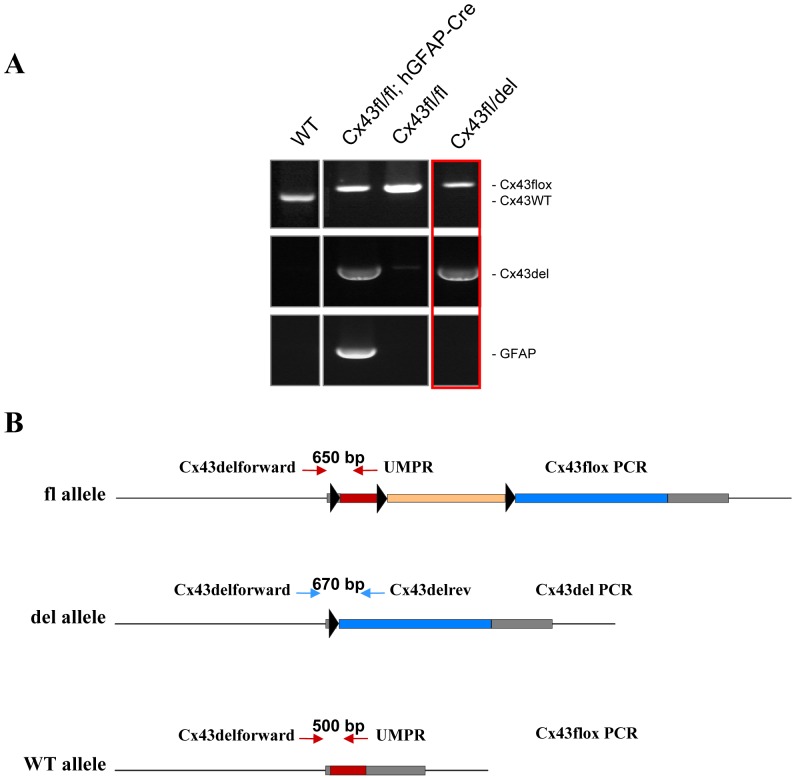
PCR for different Cx43 alleles reveals unexpected results indicative of ectopic hGFAP-Cre activity. A: Analysis of Cx43 alleles and for the presence of the hGFAP-Cre transgene by three different PCRs of offspring from Cx43^fl/fl^: hGFAP-Cre X Cx43^fl/fl^ breedings and of WT mice. The Cx43flox PCR generated a 0.5 kb WT amplicon and, in case of Cx43^fl^ alleles, a 0.65 kb amplicon. The Cx43del PCR led to a 0.67 kb amplicon (Cx43^del^ allele). Unexpectedly, we observed mice which carried a Cx43^del^ allele in the absence of the hGFAP-Cre transgene (red box). B: Scheme of Cx43 alleles. Solid boxes: Transcribed regions. Dark grey boxes: 5′ and 3′ untranslated region. Red boxes: Cx43 coding region. Yellow box: selection marker cassette. Blue box: *lacZ* reporter gene. Arrowheads: loxP sites. Arrows: primers used for Cx43flox/WT PCR (red) and Cx43del PCR (blue).

**Figure 2:**
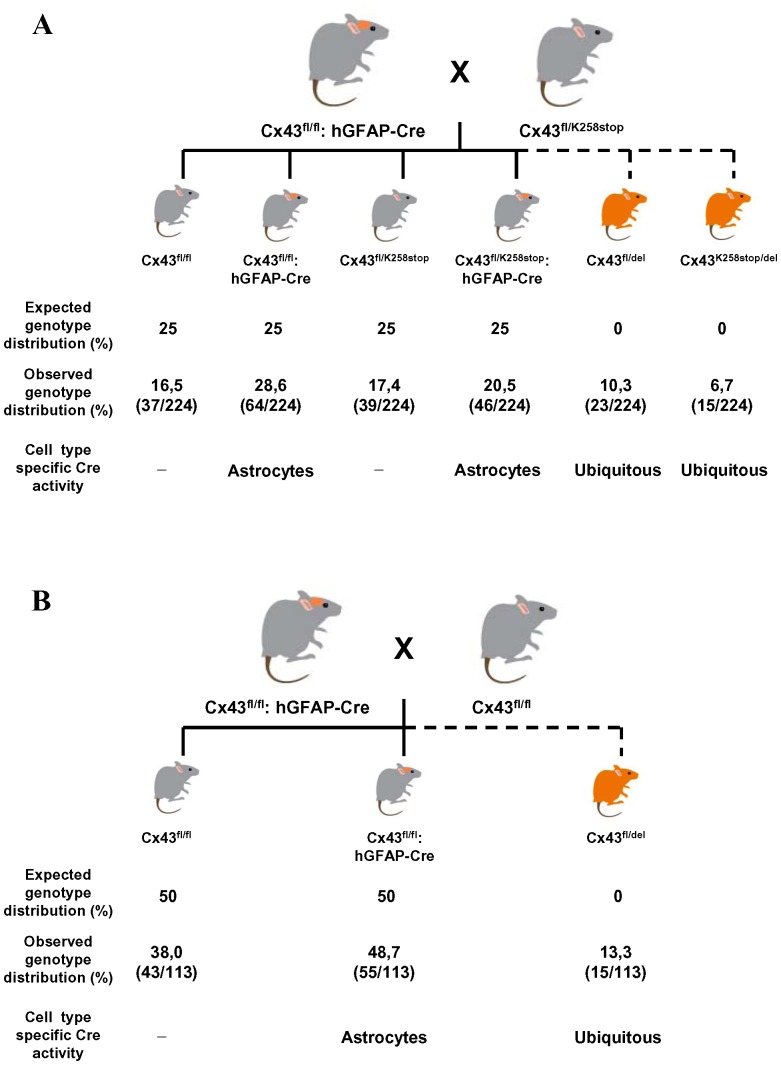
Schematic showing breeding strategies and Cx43 gene deletion using hGFAP-Cre/loxP recombination system. A: Observed progeny from homozygous Cx43^fl/fl^: hGFAP-Cre mice after mating with heterozygous Cx43^fl/K258Stop^ mice. In addition to the expected genotypes, offspring exhibiting ubiquitous Cx43 deletion was observed (indicated by the scattered line). The table below the scheme shows the expected as well as the observed genotype distribution. The number of animals per genotype of animals (n = 224) is shown in parentheses. B: Genotype analysis of the offspring from Cx43^fl/fl^: hGFAP-Cre mice mated with Cx43^fl/fl^ mice. About 50% of the progeny from this breeding were expected to be hGFAP-Cre negative. However, the number of observed Cx43^fl/fl^ mice was much lower due to ectopic Cre recombination. The number of animals per genotype of animals analyzed (n = 113) is shown in parentheses. The extent of Cx43 deletion is indicated in red.

When breeding Cx43^fl/fl^: hGFAP-Cre mice with Cx43^fl/K258Stop^ mice (irrespective of Cx30 deletion status), we obtained ‘impossible’ genotyping results from tail tip PCR, indicating the presence of a Cx43^fl^ allele in combination with a deleted floxed Cx43 allele (called ‘del’ further on), i.e. when these mice did not carry the hGFAP-Cre transgene (Fig. 1A). In order to exclude genotyping errors, we next performed a PCR specific for the hGFAP-Cre transgene and a general cre PCR and got consistent results with both PCRs (not shown). From 15 Cx43^fl/fl^: hGFAP-Cre x Cx43^fl/K258Stop^ breedings, we obtained a total of 224 mice in the offspring (Fig. 2A). In 7 of these breedings, Cre-transgenic females were used as parents, while in 8 breedings, Cre-bearing male mice were employed. A total of 114 hGFAP-Cre negative mice were among the offspring of which 38 (33%) exhibited ectopic recombination measured by the Cx43 del PCR. Of those, 34 (89%) were offspring from Cre-bearing mothers and only 4 (11%) were from Cre-transgenic fathers.

We went back to analyse possible ectopic hGFAP-Cre activity in Cx30^-/-^; Cx43^fl/fl^ x Cx30^-/-^; Cx43^fl/fl^: hGFAP-Cre breedings. A total of 58 hGFAP-Cre negative mice were among the offspring of which 15 (26%) exhibited ectopic recombination measured by the Cx43 del PCR (Fig. 2B). In 4 breedings, Cre-transgenic mothers were employed and in 7 breedings, Cre-transgenic fathers were used. Eight out of 16 Cre-negative animals from Cre-bearing mothers showed recombination (50%), while only 7 out of 42 Cre-negative offspring from Cre-transgenic fathers exhibited ectopic Cre activity (17%). We never observed mice with homozygous deletion due to the perinatal lethality of global Cx43 deletion [26], [27]. We tested if the activity was due to Cre-mediated recombination or due to spontaneous recombination of loxP sites. None out of 38 mice from Cx30^-/-^; Cx43^fl/fl^ x Cx30^-/-^; Cx43^fl/fl^ breedings (without hGFAP-Cre) was positive in the Cx43del PCR, disfavoring spontaneous recombination of loxP sites without recombinase (not shown). We conclude that the hGFAP-Cre transgene exhibited germ-line activity. Germ-line recombination occurred more often in the offspring of Cre-transgenic females.

Mice homozygous for the Cx43^K258Stop^ allele are not viable [25], similar to the perinatal lethality of the homozygous Cx43 deletion [26], [27]. However, mice carrying a Cx43^K258Stop^ allele and a Cx43 knockout allele are viable [25]. We observed that Cx43^K258Stop/del^ mice also survive when Cx30 is lacking in addition.

### Confirmation of germ-line hGFAP-Cre activity by reporter gene assays and immunostaining in the CNS

**Figure 3:**
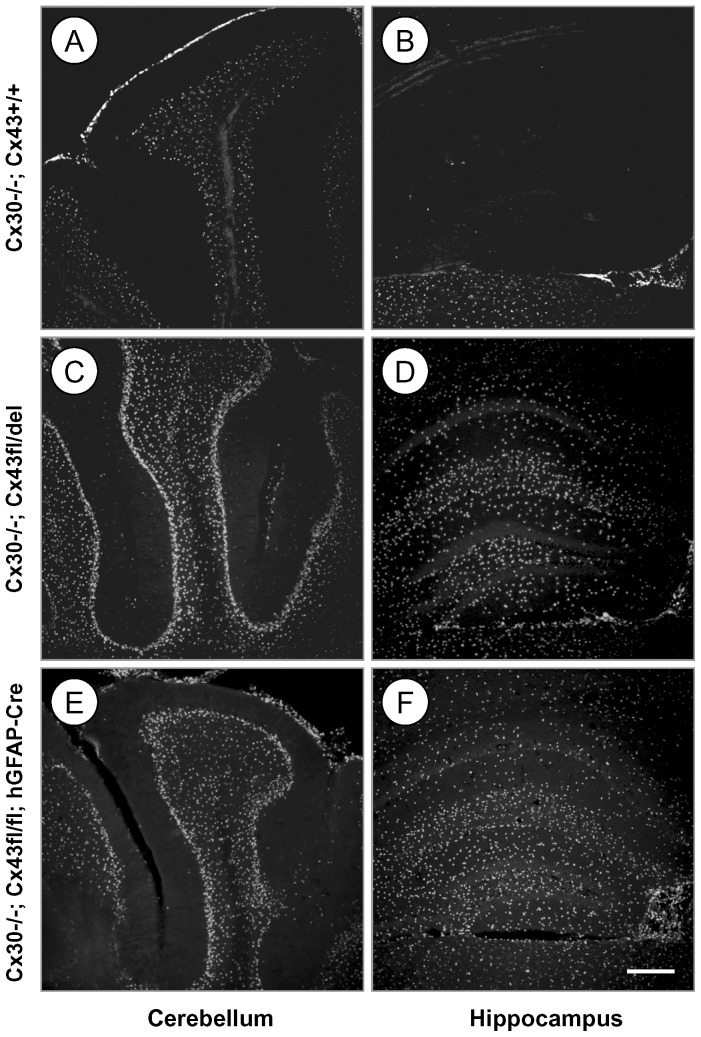
β-Gal immunoreactivity in cerebellum and hippocampus indicates ectopic activity of the hGFAP-Cre transgene. A, B: Antibody staining of Cx30^-/-^ mice shows the distribution of β-Gal expression derived from the Cx30 locus. The β-Gal expression pattern matches that of Cx30 deficient mice (see [28], [29]): Expression is moderate in the granule cell layer and leptomeninges of the cerebellum (A), and virtually absent in hippocampus (B). C, D: β-Gal staining of Cx30^-/-^;Cx43^fl/del^ mice with ectopic deletion shows much stronger β-Gal expression in cerebellum (C) and hippocampus (D) which largely matches that of DKO mice shown below. E, F: β-Gal staining of Cx30^-/-^;Cx43^fl/fl^: hGFAP-Cre mice shows the distribution of β-Gal expression derived from the Cx43 locus and the Cx30 locus. The β-Gal expression is strong both in cerebellum (E) and hippocampus (F). Bar: 200 µm.

**Figure 4:**
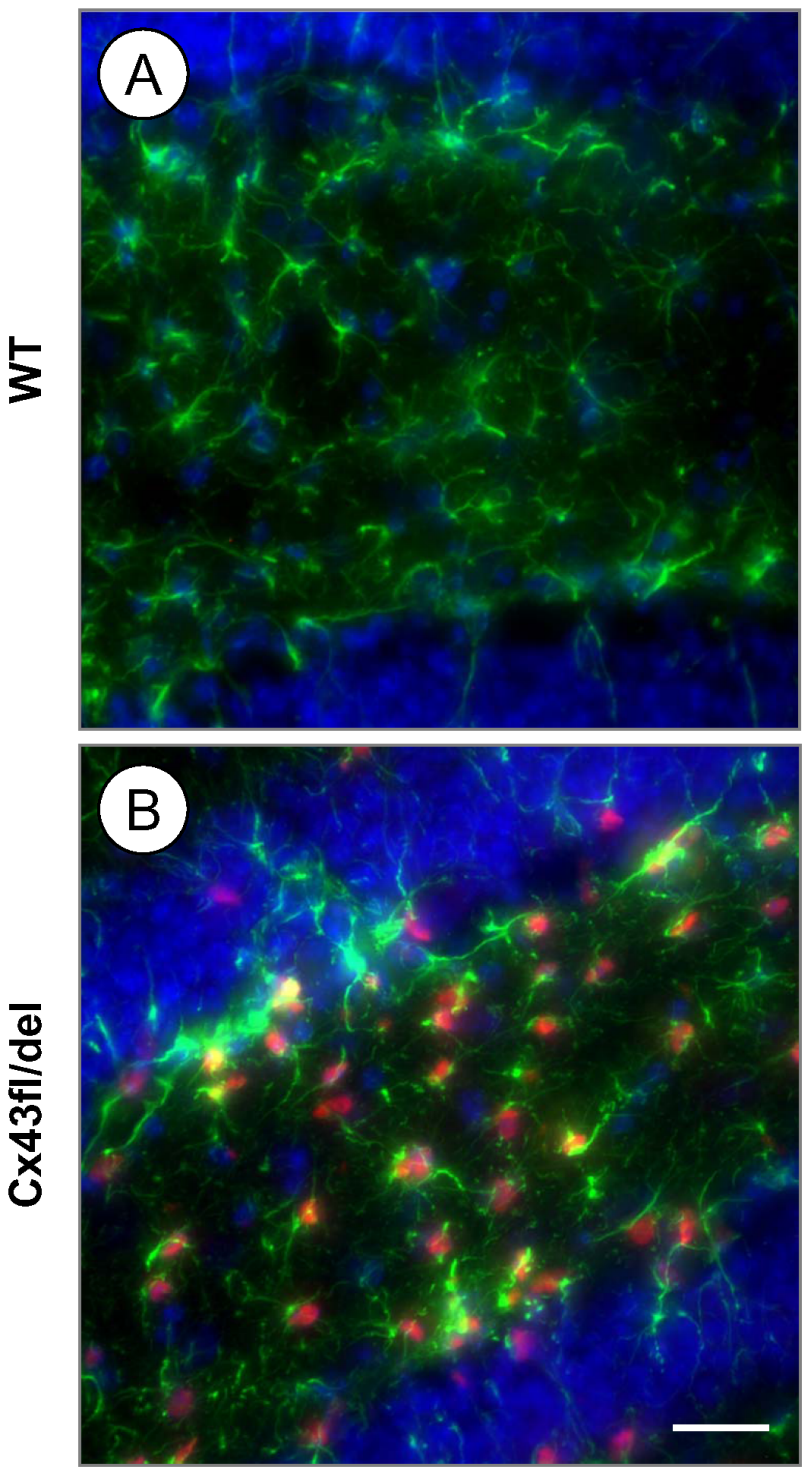
Double immunofluorescence staining for GFAP and β-Gal in the dentate gyrus. A: WT animals show absence of β-Gal expression in the dentate gyrus. B: Cx43^fl/del^ animal with ectopic deletion showing abundant β-Gal expression colocalized with the astrocytic marker GFAP similar to DKO mice. Scale bar: 50 µm.

We next evaluated lacZ reporter gene expression by immunofluorescence staining for β-Gal in the brains of Cre-negative mice, in which the Cx43del PCR indicated germ-line deletion (the deleted floxed Cx43 allele is schematically depicted in Figure 1B). Mice lacking Cx30 in all cells of the body showed β-Gal expression representing Cx30 transcription in the granule cell layer and the leptomeninges of the cerebellum (Fig. 3A), but only very weak labeling in the hippocampus (Fig. 3B; see also [28]). Cre negative mice which show germ-line deletion of one Cx43^fl^ allele (Cx43^fl/del^ mice) mediated by parental Cre expression show strongly increased labeling for β-Gal in the granule cell layer and in the Purkinje cell layer of the cerebellum (Fig. 3C), consistent with expression of Cx43 driven β-Gal in Bergmann glia [12], [29] and highly abundant expression in the hippocampus (Fig. 3D). This expression was very similar to the staining of DKO mice (Fig. 3E,F). Since β-Gal immunoreactivity from the Cx30 knockout disturbed the analysis of Cx43 driven β-Gal expression, we next tested mice which carried Cx30 WT alleles: Cx43/Cx30 WT mice were negative for β-Gal (Fig. 4A). The β-Gal immunoreactivity of deleted floxed Cx43 mice (Cx43^fl/del^ mice), i. e. which show germ-line deletion of one Cx43^fl^ allele, showed localization in cells which were positive for the astrocytic marker GFAP (Fig. 4B).

### Cx43 ablation in the absence of Cre protein

**Figure 5:**
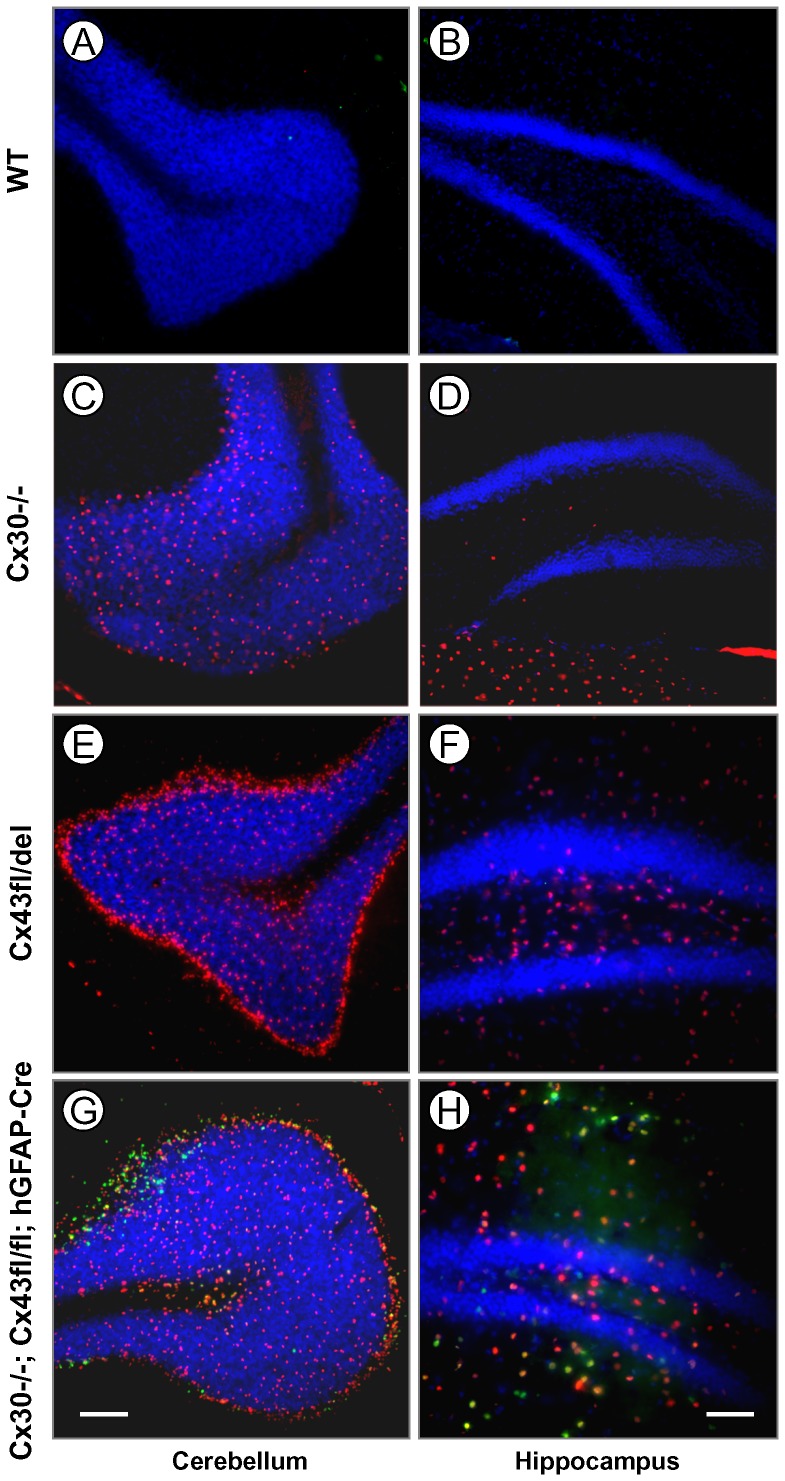
Cx43-driven β-Gal immunoreactivity occurs even in the absence of Cre immunoreactivity in mice with germ-line deletion. A–H: Immunofluorescence analysis for Cre expression (green) and β-Gal (red). Hoechst staining in blue. A, C, E, G: Cerebellum. B, D, F, H: Hippocampus. A, B: WT mice express neither β-Gal nor Cre protein. C,D: Cx30^-/-^ mice show moderate β-Gal expression in cerebellum and very weak expression in hippocampus. E, F: Cx43^fl/del^ mice exhibit strong immunoreactivity for β-Gal in both brain areas, but no Cre expression. G, H: DKO mice show β-Gal immunoreactivity essentially identical to mice with ectopic deletion and prominent expression of Cre in both brain areas. Bar: 100 µm (50 µm for F and H).

Next, we correlated Cre expression with Cx43 ablation *in situ* by immunofluorescence detection of Cre recombinase and of β-Gal in cerebellar and hippocampal cryosections. WT mice were negative both for β-Gal and Cre (Fig. 5A,B). Mice lacking Cx30 showed Cx30 driven β-Gal expression in the cerebellum, very weak labeling in the hippocampus and were likewise Cre negative (Fig. 5C,D). Mice, which experienced ectopic deletion of one Cx43^fl^ allele showed strong immunoreactivity for β-Gal, but lacked Cre immunoreactivity (Fig. 5E,F). Immunoreactivity for β-Gal was very similar in DKO mice lacking both Cx43 and Cx30, which showed robust Cre immunoreactivity (Fig. 5G,H), in contrast to the mice with ectopic deletion (Fig. 5E,F).

**Figure 6:**
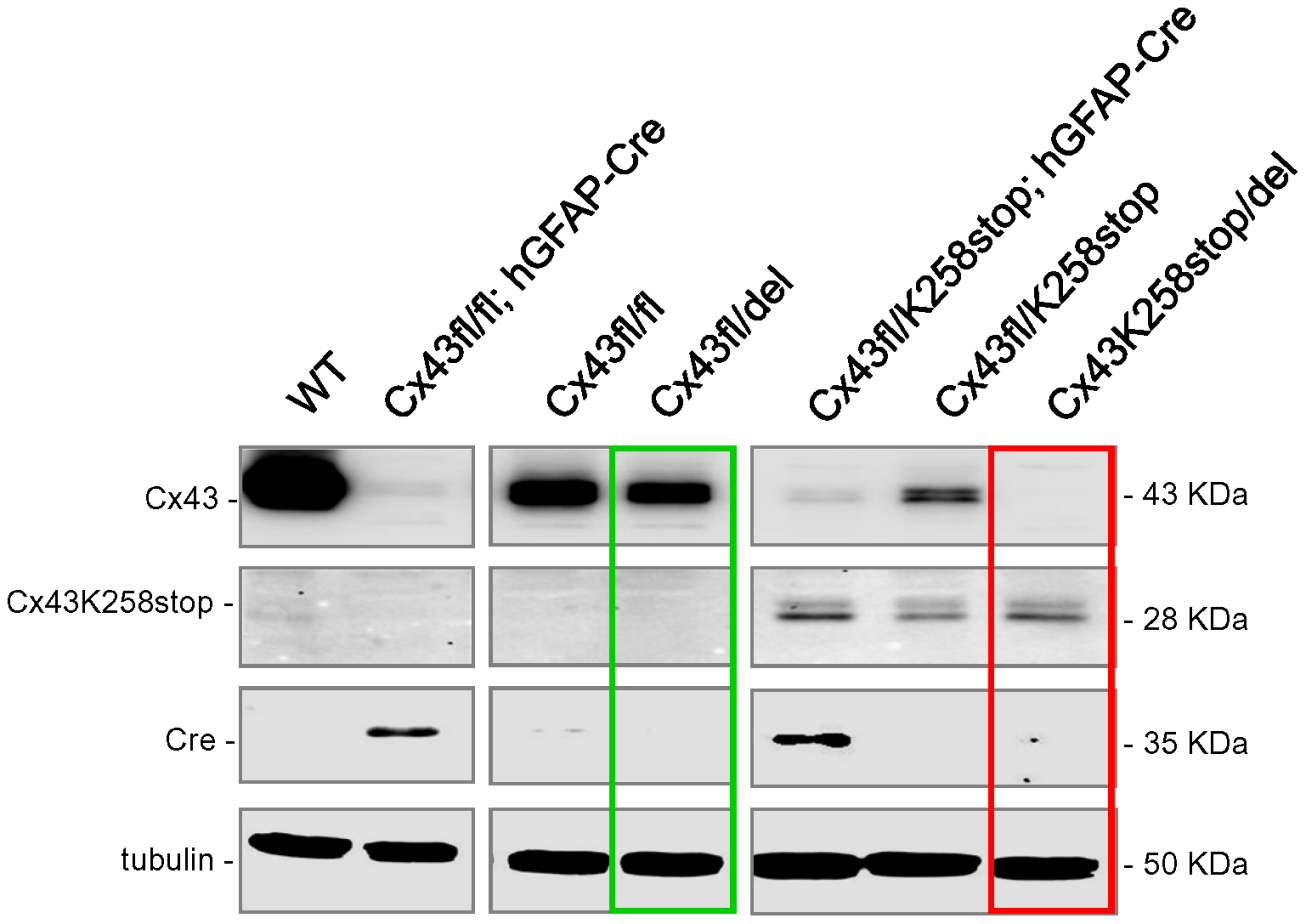
Immunoblot analysis of hippocampal lysates for Cre and Cx43 protein indicates loss of Cx43 expression in the absence of Cre protein. In Cx43^fl/del^ mice (green box), immunoreactivity for Cx43 is reduced by about 50% compared to Cx43^fl/fl^ mice. Immunoreactivity for the full length Cx43 is completely lost in Cx43^K258Stop/del^ mice (red box). Please note absence of Cre immunoreactivity in the Cx43^fl/del^ and Cx43^K258Stop/del^ lanes, consistent with negative PCR results for hGFAP-Cre and internal Cre PCRs. Upper row: Cx43 immunoreactivity at 43 kDa (full length protein, Cx43) with an antibody directed to the C-terminus. Second row: Cx43 immunoreactivity at 28 kDa (truncated protein, Cx43K258stop) with an antibody directed to the N-terminus. Third row: Immunoreactivity for the Cre recombinase (Cre). Fourth row: Tubulin loading control. WT: Cx43^+/+^. kDa: Kilodalton.

Immunoblot analysis of hippocampal lysates confirmed that Cx43^K258Stop/del^ mice with germ-line deletion of Cx43 indeed have lost immunoreactivity for the full length Cx43 (the Cx43 antibody used detected the C-terminus of Cx43, which is lacking in the truncated Cx43^ K258Stop^ form). WT mice and mice carrying Cx43^fl^ alleles show Cx43 expression with an antibody directed to the C-terminus at a Mw of 43 kDa (upper row), but lack immunoreactivity for an N-terminal Cx43 antibody at a Mw of 28 kDa (corresponding to the Cx43K258Stop protein; second row). Cx43^fl/fl^: hGFAP-Cre mice show strongly decreased expression of Cx43 at 43 kDa, corresponding with their immunoreactivity for Cre in hippocampal lysates (third row). Residual Cx43 protein levels in DKO samples are due to the remaining expression of Cx43 in cell types not targeted by hGFAP-Cre, such as endothelial cells and leptomeningeal cells (see [12]). Cx43^fl/fl^ mice show less abundant Cx43 protein expression compared to WT mice due to the targeted modification of the Cx43 locus, as already reported [26]. Consistently, in deleted floxed Cx43 mice (Cx43^fl/del^ mice; green box in Fig. 6), immunoreactivity for Cx43 is further reduced by about 50% due to loss of one Cx43^fl^ allele in spite of the absence of Cre protein. Mice carrying the Cx43^K258Stop^ allele show immunoreactivity for the 28 kDa truncated protein with the N-terminal antibody and, depending on the presence of a Cx43^fl^ allele, immunoreactivity for the full length Cx43 protein at 43 kDa. Cx43^fl/K258Stop^: hGFAP-Cre mice show strongly decreased immunoreactivity for the full length Cx43 protein, corresponding with immunoreactivity for Cre, while the levels of the truncated protein are not changed compared to Cx43^fl/K258Stop^ mice lacking Cre. No Cre protein is expressed, but immunoreactivity for the full length Cx43 is completely lost in deleted floxed Cx43 (Cx43^K258Stop/del^ mice; red box in Fig. 6). We here demonstrate with *in situ* immunolocalization and immunoblotting that ectopic activity occurs frequently in Cx43^fl/fl^ x Cx43^fl/fl^: hGFAP-Cre breedings and in Cx43^fl/K258Stop^ x Cx43 ^fl/fl^: hGFAP-Cre breedings. This phenomenon was evident from the ubiquitous deletion of Cx43 observed in offspring devoid of Cre recombinase, indicating germ-line recombination mediated by hGFAP-Cre.

### Assessment of germ-line hGFAP-Cre activity in heart and brain

**Figure 7:**
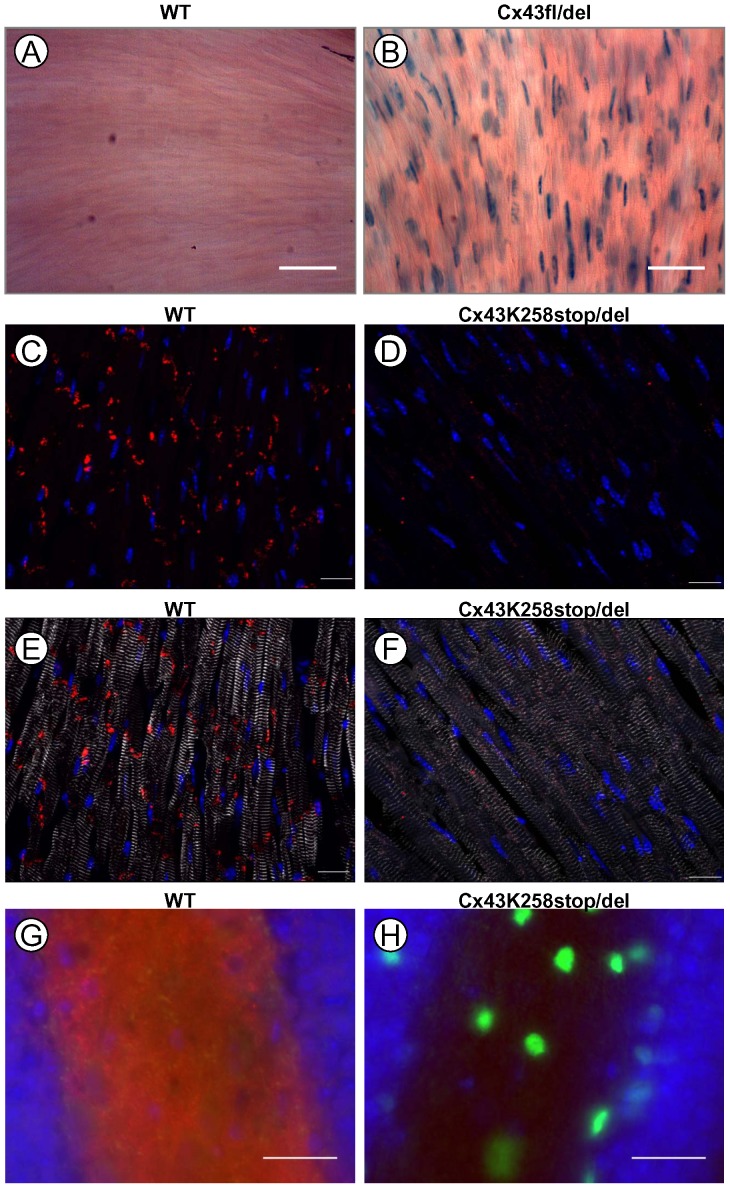
Ectopic activity of the hGFAP-Cre transgene in the heart and brain. A, B: X-Gal staining of sections from the left ventricle. WT mice do not show X-gal staining (A), while Cx43^fl/del^ mice derived from Cx43 ^fl/fl^: hGFAP-Cre x Cx43^fl/K258Stop^ breedings exhibit β-Gal activity which is confined to the nucleus of cardiomyocytes (B). C, D and E, F: Antibody staining of sections from the left ventricle with an antibody directed to the 20 C-terminal amino acids of Cx43 (red) together with Hoechst nuclear stain (blue). E and F show additionally an antibody directed to sarcomeric α-actinin (white). WT mice show prominent labelling of gap junctional plaques at intercalated disks between cardiomyocytes (C and E), while Cx43^K258Stop/del^ mice lack immunoreactivity, consistent with absence of the Cx43 C-terminus (D and F). G, H: Triple staining for the Hoechst nuclear stain (blue), β-Gal (green) and the C-terminal epitope of Cx43 (red). WT mice show prominent Cx43 expression in the hilus of the dentate gyrus and no β-Gal expression (G). By, contrast, Cx43^K258Stop/del^ mice do not show immunoreactivity for the C-terminal epitope of Cx43 but show strong β-Gal expression, consistent with deletion of the Cx43^fl^ allele (H). Bar: 50 µm for A and B, 20 µm for C-F and 25 µm for G and H.

Besides astrocytes and leptomeningeal cells in the CNS, Cx43 is also prominently expressed in the heart [27], and likewise Cx43-driven β-Gal reporter expression has been demonstrated in the heart [26]. Any germ-line activity mediated by hGFAP-Cre in the zygote or early embryo should therefore as well lead to recombination in the adult heart, an organ which is not targeted by hGFAP-Cre [11]. We therefore investigated heart sections of mice with germ-line deletion of Cx43 for β-Gal activity by X-gal staining (Fig. 7). WT mice lack β-Gal (Fig. 7A). By contrast, hGFAP-Cre negative offspring from Cx43^fl/fl^: hGFAP-Cre x Cx43^fl/K258Stop^ breedings carrying the deleted floxed Cx43 (Cx43^fl/del^) genotype, i.e. showing germ-line deletion, exhibited prominent β-Gal activity in the heart visualized by X-gal staining which was localized to the nucleus (the engineered β-Gal contained a nuclear localization signal). We also assessed Cx43 immunoreactivity in heart sections of Cx43^K258Stop/del^ mice from the same breedings, using an antibody directed to the 20 C-terminal amino acids of Cx43, which are lacking in the truncated variant of Cx43. While we obtained typical labeling of gap junction plaques in the intercalated discs between ventricular cardiomyocytes of WT mice (Fig. 7C,E; [25], we did not observe any labeling in heart sections of Cx43^K258Stop/del^ mice using this antibody (Fig. 7D,F), confirming germ-line deletion of full length Cx43 by parental hGFAP-Cre protein. Similarly, we observed loss of immunoreactivity for the C-terminal epitope of Cx43 concomitant with gain of β-Gal immunoreactivity in the hippocampus of Cx43^K258Stop/del^ mice when compared to WT mice (Fig. 7G,H).

### Germ-line deletion mediated by the nestin-Cre transgene

**Figure 8:**
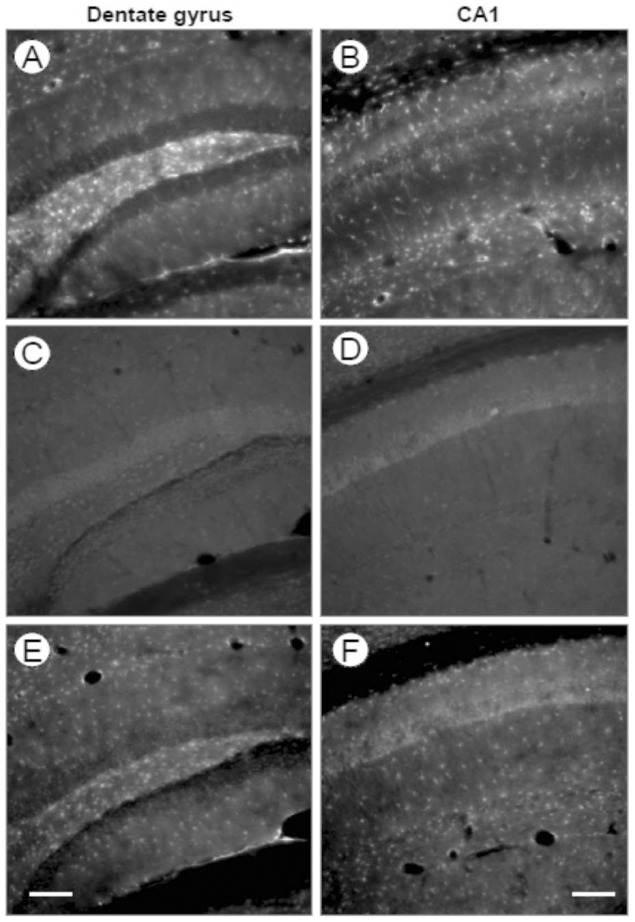
GFP immunoreactivity in hippocampal dentate gyrus and CA1 region indicates ectopic activity of the nestin-Cre transgene. A, B: Chicken anti-GFP antibody staining of Cx30^-/-^; Cx43^flG138R/flG138R^: nestin-Cre mice shows the distribution of EGFP expression under control of Cx43 regulatory elements. Expression is stronger in the dentate gyrus (A), especially in the hilus, compared to the CA1 region (B). C, D: Cx30^-/-^; Cx43^flG138R/flG138R^ mice exhibit no immunoreactivity for GFP in both hippocampal regions. E, F: Cx30^-/-^; Cx43^flG138R/flG138R^ mice with germ-line activity of nestin-Cre in the parents show GFP staining in the dentate gyrus (E) and the CA1 region (F) with similar density as in Cx30^-/-^;Cx43^flG138R/flG138R^: nestin-Cre mice. Bar: 100 µm.

We have recently investigated conditional knock-in mice with a replacement of WT Cx43 by the Cx43G138R point mutation [22] directed to astrocytes via a nestin-Cre transgene [20]. In these mice, Cre-mediated recombination leads to expression of the Cx43G138R point mutation together with EGFP. Germ-line nestin-Cre activity in Cx43G138R point mutated mice was assessed by negativity for both the nestin -Cre PCR and the internal cre PCR (data not shown) and GFP immunostaining (Fig. 8A–F). In 4 out of 14 Cre-negative mice investigated, we observed EGFP-reporter expression in the hippocampus, indicating ectopic deletion mediated by nestin-Cre (Fig. 8E,F). Thus, these data indicate that germ-line Cre activity occurs in the CNS-restricted nestin-Cre transgene as well.

## Discussion

Here we describe spontaneous germ-line recombination activity of an hGFAP-Cre transgene [11] and of a nestin-Cre transgene [20] when bred to Cx43 conditional alleles. Following the initial characterization and confirmation of faithful recombination mediated by hGFAP-Cre, we kept mice with astrocyte-specific deletion in a homozygous floxed state for several years. Global homozygous deletion of Cx43 in all cells of the body is perinatally lethal due to morphological disturbances of the right ventricular outflow tract [26], [27], whereas deletion of one allele in all cells of the body does not result in an overt phenotype. We now observed ectopic activity of hGFAP-Cre in an allelic setting compatible with survival of global Cx43 deletion (i.e. in combination with the Cx43^K258Stop^ allele; [25]). The germ-line activity mostly occurred in the offspring of Cre-transgenic females and was not linked to hGFAP-Cre transgene transmission to the offspring. The offspring of Cre-transgenic males was less affected. Although the exact mechanism responsible for hGFAP-Cre germ-line activity remains elusive, the occurrence of germ-line deletion can be minimized by using Cre-bearing fathers. However, our data clearly show that the GFAP promoter is active during various stages of embryonic development in progenitor cells with a rather broad differentiation potential. We see the same phenomenon in nestin-Cre mice. Such an inheritance pattern of ectopic recombination even in Cre-negative offspring is already known from the PGK-Cre transgene [30], a Keratin5-Cre transgene [31], and an αMHC-Cre line [7]. Once we have observed germ-line recombination activity of hGFAP-Cre in combination with the Cx43^K258Stop^ allele, we tested our Cx43^fl/fl^: hGFAP-Cre colony and frequently found germ-line deletion of single floxed alleles. Cx43 protein expression from a Cx43^fl^ allele is already rather low (∼50% of a WT allele). The expression of a ‘control’ mouse with hidden germ-line deletion of one Cx43^fl^ allele is thus minimally different from a full Cx43 knockout compared to a Cx43^fl/fl^ animal with two copies of a floxed allele. Compared to lack of hGFAP-Cre activity giving rise to pseudo KO mice [10], the germ-line hGFAP-Cre and nestin-Cre activity we show here is much more frequent.

Our data clearly indicated that germ-line recombination has occurred. First, the deletion was present in all investigated organs (brain, heart, tail and also testes and ovaries). Deletion cannot occur at later stages of embryogenesis as this would result in chimeras, which we never observed. Second, the incidence of germ-line deleted animals was depended on the maternal or paternal inheritance of the Cre transgene. The event is not abundant, as only a minority of Cre-negative animals inherited a deleted floxed allele. Third, recombination after fertilization is unlikely to occur since Cre would then excise both floxed alleles, resulting in a Cx43^del/del^ genotype, which is lethal. Fourth, recombination was not due to auto-excision as breedings with Cre-negative parents never had any deleted recombined offspring. To identify potential Cre-induced germ cell recombination, we used β-Gal immunostaining in combination with a germ cell-specific marker (VASA). In male and female germ-line deleted Cx30^+/-^; Cx43^fl/del^ and Cx30^+/+^; Cx43^fl/del^ animals at all time points investigated spermatogonia, spermatocytes, spermatids (and sperm) as well as all stages of present oocyte development remained immunonegative for β-Gal (not shown). Although these cells express Cx43 during differentiation, the expression level might be too low to generate sufficient β-Gal for immunohistochemical detection. Therefore we additionally tried to directly monitor Cre protein in germ cells using different antibodies directed against Cre, but we failed (not shown). The reasons for these negative results remain unclear. We suspect that either our different protein detection assays were too insensitive to monitor germ line deletion, or the deletion occurred at another time point in development than those investigated here. Moreover, epigenetic changes cannot be excluded [31], [32]. Using a more ubiquitous reporter such as floxed ROSA26 would probably better reveal the broad recombination potential of hGFAP-Cre and nestin-Cre mice.

A rigid quality control of mice with astrocyte-directed gene inactivation is required to exclude germ-line deletion by hGFAP-Cre and nestin-Cre within transgenic colonies. We here present strategies for pre- and post-experimental assessment of astrocyte-directed Cx43 KO mice by DNA analysis, immunohistochemistry and immunodetection and would like in the following to shortly explain why PCR analysis is the most straight forward approach to identify germ line deleter mice. Deletion status measured by the Cx43del PCR for deletion of the Cx43^fl^ allele in tail tip DNA was consistent with the deletion status in brain and heart measured by reporter gene expression, immunoblotting and immunofluorescence. We found that PCR analysis of tail-tip DNA is suited to detect hGFAP-Cre negative mice, which experienced germ-line deletion. Thus, the tail-tip genotyping allows pre-experimental assessment of animals in order to estimate the deletion status of Cx43^fl^ alleles in mice lacking the hGFAP-Cre or nestin-Cre transgenes. In case of a lack of reporter genes, PCR for detection of a deleted floxed allele should be employed. Since germ-line deletion also occurs in the presence of hGFAP-Cre and nestin-Cre, it is not possible to distinguish clearly between normal deletion and germ-line deletion in the tail of hGFAP-Cre positive mice. Post-experimental screening for β-Gal expression in the heart is required to exclude germ-line recombination of the Cx43^fl^ allele, as we showed here exemplary for hGFAP-Cre positive mice.

Several other GFAP-Cre transgenic mice have been generated using identical or similar hGFAP promoter elements [33]–[36]. Since a recent report indicated that a tamoxifen-inducible RIP-CreER transgene for timed recombination in beta cells of the pancreas is active even without inducer [37], ectopic activity of hGFAP-Cre may even constitute a problem for inducible gene switches employing hGFAP promoter elements [38], [39]. Our findings on germ-line recombination of the hGFAP-Cre and nestin-Cre transgenes are highly relevant to other groups working in the field of astroglial gene function, since fate mapping approaches as well as phenotype analysis could be seriously flawed by the lack of a cell-specific expression pattern.

Especially if global deletion of a floxed gene is not lethal, the mouse colony may, with time, become contaminated by global knockouts. We therefore recommend to closely monitor the activity status of GFAP-Cre and of nestin-Cre transgenic lines.

## Materials and Methods

### Statement on the ethical treatment of animals

Maintenance and handling of animals used in this study was according to local government regulations. Experiments have been approved by the State Office of North Rhine-Westphalia, Department of Nature, Environment and Consumerism (LANUV NRW, approval number 9.93.2.10.31.07.139). All measures were taken to minimize the number of animals used.

### Animals

The development and genotyping of transgenic mice used in this work has been previously reported. Cx43^fl^ mice (Gja1^tm1Kwi^) carry a floxed Cx43 coding region. Cre-mediated recombination leads to expression of an embedded *lac*Z gene encoding a nuclear β-Gal; [26]. Cx43^del^ mice (Gja1^tm1.1Kwi^) carry a *lac*Z gene (encoding a nuclear β-Gal) in place of the Cx43 coding region [26]. The hGFAP-Cre mice (Tg(GFAP-cre)25Mes; [11]) expressing Cre also in neuroglial progenitor cells during development were used for astrocyte-directed deletion of Cx43 [12], [13]. Cx30^-^ mice (Gjb6^tm1Kwi^) carry a lacZ gene encoding β-Gal fused to a nuclear localization signal (NLS) in place of the Cx30 coding region [24]. Cx43^K258stop^ (Gja1^tm4Kwi^) mice carry a truncated Cx43 coding region in which the codon for lysine at amino acid position 258 was replaced by a stop codon [25]. Most Cx43^K258stop^ homozygous mice die within the first 5 days after birth due to a defect of the epidermal barrier, but Cx43^K258stop/del^ compound heterozygous mice harboring one C-terminally truncated Cx43 and one Cx43 knockout allele reach adulthood [25], [40]. Cx43^flG138R^ (Gja1^tm8Kwi^) mice express the human point mutation Cx43G138R following cre activity instead of WT Cx43. As reporter gene in these mice, EGFP is expressed along with the Cx43G138R point mutation by way of an IRES element [22]. Nestin-Cre (Tg(Nes-cre)1Kln/J) mice [20] were used to delete WT Cx43 and replace its expression by the point mutation Cx43G138R.

### Genotype Analysis by PCR

Genomic tail tip DNA was prepared as described previously [26]. For routine genotypic analysis, genomic DNAs from tail biopsies were used for PCR with different primers. For detection of both the hGFAP-Cre and nestin-Cre transgenes, an internal cre PCR was applied. Primers intcreup (5′-TTT GCC TGC ATT ACC GGT CGA TGC-3′) and intcrerev (5′-TCC ATG AGT GAA CGA ACC TGG TCG-3′) were used, generating a 400 bp amplicon of part of the Cre-coding region. In addition, the hGFAP-Cre PCR [11] and Nestin-Cre PCR [20] were applied. For simultaneous detection of the Cx43 floxed (Cx43^fl^) allele and the *Cx43* WT (*Cx43^+^*) allele, primers UMPR (5′-TCA CCC CAA GCT GAC TCA ACC G-3′) and Cx43delforward (5′-GGC ATA CAG ACC CTT GGA CTC C-3′) were applied (Cx43flox PCR), generating a 650 bp Cx43^fl^ amplicon and a 500 bp WT amplicon, spanning the junction between the intron of *Cx43* and the *Cx43* coding region. For detection of the deleted *Cx43* (*Cx43^del^*) allele, primers Cx43delforward and Cx43delrev (5′-TGC GGG CCT CTT CGC TAT TAC G-3′) were used (Cx43del PCR), generating a 670 bp amplicon of the junction between the intron of *Cx43* and the β-Gal coding region. For genotyping of Cx43^K258Stop^ mice, primers delCT-HO (5′- GCA TCC TCT TCA AGT CTG TCT TCG -3′) and RO-delCT (5′- CAA AAC ACC CCC CAA GGA ACC TAG -3′) were applied, resulting in a 851 bp amplicon for the Cx43^+^ allele and a 452 bp amplicon for the Cx43^K258Stop^ allele as described previously [25]. Genotyping of Cx43^flG138R^ mice was performed by using primers polyAforward (5′-GGG GGT GAA GGA GTT TTC AGC AGT GC-3′), loxPforward (5′-GCA CTT GGT AGG TAG AGC CTG TCA GGT C-3′) and a primer binding in the Cx43 coding region (5′-GCT TCC CCA AGG CGC TCC AGT CAC CC-3′), generating a 400 bp Cx43floxed amplicon and a 350 bp WT amplicon.

### Immunohistochemistry and histochemical staining

The lacZ gene encodes the enzyme β-Gal, which converts the colourless dye X-gal into a blue stain. X-gal stainining of heart sections was performed according to [26].

For double immunofluorescence stainings, mice were transcardially perfused with 4% paraformaldehyde (PFA), brains and hearts were removed, and after fixation in 4% PFA, and cryoprotection using 30% sucrose in PBS, 40 µm cryostat sections were cut (Microm HM560, Walldorf, Germany). Sections were blocked with 5% NGS (Normal Goat Serum Dako Germany) and 0.3% Triton X-100 (Sigma, Munich, Germany) in PBS for 2 h at room temperature. Sections were incubated overnight with primary antibodies (5% NGS and 0.1% Triton X-100). After washing, sections were incubated with secondary antibodies and washed again. As primary antibodies, rabbit polyclonal anti-β-Gal antibodies (1∶500 Molecular Probes, Leiden, The Netherlands) were used. As secondary antibodies, goat anti-rabbit antibodies conjugated to Alexa fluor 594 (1∶500 A11037, Invitrogen) were applied. Mouse monoclonal anti-GFAP antibodies (1∶500, MAB360, Chemicon) and the secondary antibodies, goat anti-mouse antibodies conjugated to Alexa fluor 488 (1∶300, A11029, Invitrogen) were applied for detection of GFAP. For detection of Cre-recombinase expression, a mouse monoclonal anti-Cre recombinase antibody (1∶500, MAB3120, Chemicon) was used as a primary antibody. As secondary antibodies, goat anti-mouse antibodies conjugated to Alexa fluor 488 (1∶300, A11029, Invitrogen) were applied. Sections were stained subsequently with Hoechst 33342 (0.25 mg/ml, Molecular Probes). For detection of Cx43flG138R expression, a chicken polyclonal anti-GFP antibody (1∶500, ab13970, Abcam) was used as a primary antibody. As secondary antibodies, goat anti-chicken antibodies conjugated to Alexa fluor 488 (1∶300, A11039, Invitrogen) were used.

Images were taken with a digital SPOT camera (Diagnostic Instruments, Sterling Heights, MI) and MetaView software (Universal Imaging, West Chester, PA), using a Zeiss Axiophot equipped with fluorescence optics. Several optical sections through the depth of the slice were digitally combined to yield the final images.

Immunofluorescence analyses on mouse hearts were carried out on 10 µm cryosections of adult left ventricular tissue. Hearts were harvested, perfused with PBS, fixed with 4% paraformaldehyd (PFA); hearts were washed after 24 h and transferred to 20% sucrose solution for cryopreservation. Cryosections were permeabilized with 0.2% Triton X-100 in PBS. For immunostaining, slices were incubated overnight at 4°C with primary antibodies diluted in 5% donkey serum in PBS. Nonspecific binding sites were blocked with 5% donkey serum. As primary antibodies, α-actinin (1∶400, Sigma-Aldrich Chemie GmbH, München, Germany) and Cx43 (1∶2000) were used; the Cx43 antibody is directed against the C-terminus and was generated by Peptide Specialty Laboratories GmbH, Heidelberg, Germany. Secondary antibodies conjugated to DyLight-549 or Cy5 (1∶400, Dianova GmbH, Hamburg, Germany) as well as staining of nuclei with Hoechst 33342 (1∶1000, Sigma-Aldrich Chemie GmbH) were used. Pictures were taken with an Axio Observer.Z1 equipped with an ApoTome and AxioCamMR (Carl Zeiss AG, Oberkochen, Germany); images were acquired with the Zeiss software AxioVision. Identical exposure times for acquisition of Cx43 positive and negative slices were employed.

### Preparation of brain lysates, immunoblotting and data evaluation

Hippocampal tissue was removed and quick-frozen in liquid nitrogen. The hippocampal lysates were prepared in modified RIPA lysis buffer (50 mM Tris, 150 mM NaCl, 0.5% Nonidet P40, 0.5% Na-DOC, 1% Triton X-100, 0.5% SDS) supplemented with Roche Complete Mini protease inhibitor cocktail, 1 tablet/10 ml (Roche, Mannheim, Germany) In brief, the tissue was homogenized with a plastic pestle in a 1.5 ml tube in the lysis buffer, then disrupted with a 27 gauge needle and supersonic slat (until homogeneous) and incubated on ice for ∼30 min. Supernatants were collected after 30 min centrifugation at 13,000 x g at 4°C. Total protein content was assayed with BCA (Pierce, Bonn, Germany) and 50 µg of total protein per lane was used. Lysates were mixed with sample buffer (62.5 mM Tris-Cl, pH 6.8, 3% SDS, 0.01% bromophenol blue, 5% β-mercaptoethanol, 10% glycerol) and heated for 10 min at 65°C. Proteins were separated with standard SDS-PAGE in denaturing conditions and electroblotted onto a PVDF membrane. Membranes were blocked with 5% milk powder in TBS (pH 7.4) containing 0.05% Tween-20 and incubated O/N at 4°C on a rotator with primary antibodies: rabbit polyclonal anti-Cx43 (1∶5000, Sigma, Steinheim, Germany), mouse monoclonal anti-Cx43NT (1∶200, Fred Hutchinson Cancer Research Center (FHCRC), Seattle, USA), rabbit polyclonal anti-Cre (1∶1000, Merck, Darmstadt, Germany), mouse monoclonal anti-α-tubulin (1∶20,000, Sigma, Steinheim, Germany). Secondary antibodies used: goat-anti-mouse HRP conjugate (1∶10,000, GE Healthcare, Little Chalfont Buckinghamshire, UK) goat-anti-rabbit HRP conjugate (1∶10,000, GE Healthcare). All antibodies, including secondary antibodies, were diluted in 5% milk powder in TBS (pH 7.4) containing 0.05% Tween-20, except for the mouse monoclonal anti-Cx43NT antibody, which was diluted in 1% milk powder in TBS (pH 7.4). Equal loading of the lanes was confirmed by α-tubulin staining of the same membrane. For stripping, Pierce “Restore” stripping buffer was used for all blots. Membranes were usually re-blocked after stripping for 2 h at room temperature. For visualisation of HRP, the West Dura substrate (Pierce) was used and chemiluminescence was detected with the Gene Gnome digital documentation system (Synoptics, Cambridge, UK).
